# The Effects of Reducing Preparation Time on the Execution of Intentionally Curved Trajectories: Optimization and Geometrical Analysis

**DOI:** 10.3389/fnhum.2017.00333

**Published:** 2017-06-29

**Authors:** Dovrat Kohen, Matan Karklinsky, Yaron Meirovitch, Tamar Flash, Lior Shmuelof

**Affiliations:** ^1^Department of Computer Science and Applied Mathematics, Weizmann Institute of ScienceRehovot, Israel; ^2^Computer Science and Artificial Intelligence Laboratory (CSAIL), Massachusetts Institute of Technology (MIT)Cambridge, MA, United States; ^3^Department of Brain and Cognitive Sciences, Ben-Gurion University of the NegevBeer Sheva, Israel; ^4^Department of Physiology and Cell Biology, Ben-Gurion University of the NegevBeer Sheva, Israel; ^5^Zlotowski Center for Neuroscience, Ben-Gurion University of the NegevBeer Sheva, Israel

**Keywords:** reaching, optimization, reaction time, obstacle avoidance, drawing, motor planning

## Abstract

When subjects are intentionally preparing a curved trajectory, they are engaged in a time-consuming trajectory planning process that is separate from target selection. To investigate the construction of such a plan, we examined the effect of artificially shortening preparation time on the performance of intentionally curved trajectories using the Timed Response task that enforces initiation of movements prematurely. Fifteen subjects performed obstacle avoidance movements toward one of four targets that were presented 25 or 350 ms before the “go” signal, imposing short and long preparation time conditions with mean values of 170 ms and 493 ms, respectively. While trajectories with short preparation times showed target specificity at their onset, they were significantly more variable and showed larger angular deviations from the lines connecting their initial position and the target, compared to the trajectories with long preparation times. Importantly, the trajectories of the short preparation time movements still reached their end-point targets accurately, with comparable movement durations. We hypothesize that success in the short preparation time condition is a result of an online control mechanism that allows further refinement of the plan during its execution and study this control mechanism with a novel trajectory analysis approach using minimum jerk optimization and geometrical modeling approaches. Results show a later agreement of the short preparation time trajectories with the optimal minimum jerk trajectory, accompanied by a later initiation of a parabolic segment. Both observations are consistent with the existence of an online trajectory planning process.Our results suggest that when preparation time is not sufficiently long, subjects execute a more variable and less optimally prepared initial trajectory and exploit online control mechanisms to refine their actions on the fly.

## Introduction

During the process of planning a reaching movement, subjects select a goal for their action (Wolpert and Landy, 2012) and are subsequently engaged in motion planning; either in the form of planning a specific trajectory (Flash and Hogan, 1985; Ganesh et al., 2010) or by designing an optimal control policy, that allows for on-line feedback corrections (Todorov and Jordan, 2002). Accumulating results have suggested that when subjects perform point-to-point movements in the absence of any additional spatial constraints, the planning time is primarily affected by target selection, which may involve the representation of multiple options (Cisek and Kalaska, 2005), and motor planning is likely to rapidly occur before movement initiation (Wong et al., 2016). However, when task execution includes additional spatial constraints (such as the need to avoid an obstacle or to draw a predefined shape); motion planning is more complex, naturally requiring additional planning resources. Indeed, it was shown that trajectory planning under these conditions requires additional preparation time (Wong et al., 2016), and may raise the need for constructing the trajectory from several distinct movement segments (Morasso and Mussa Ivaldi, 1982). It was also suggested that the generation of curved trajectories involves the activation of different cortical areas than those participating in the planning of straight point-to-point movements (Schwartz, 1994).

The processes underlying action planning have traditionally been investigated using reaction time tasks (Hyman, 1953; Simon and Rudell, 1967; Donders, 1969; Philipp and Koch, 2011; Boisgontier et al., 2014). It was shown that the durations of the reaction time intervals depend on the intensity, duration and modality of the sensory stimulus presented to the subject (Todd, 1912; Simon et al., 1971; Ulrich et al., 1998), as well as on the number of the possible motor responses (Hick, 1952), and the complexity of the required inter-limb coordination patterns (Philipp and Koch, 2011; Boisgontier et al., 2014). Furthermore, it was shown that when target selection involves perceptual grouping, a trade-off between reaction time and online trajectory corrections arises, suggesting that while movement onset and target selection processes are related, they can be decoupled (Song and Nakayama, 2008), and that reaction time tasks may overlook online planning components. The concern that reaction time durations do not adequately reflect the cognitive processes underlying motor planning was further supported by a recent study showing that even when subjects are forced to move early, compared to their natural reaction time, they are still capable of producing accurate movements (Haith et al., 2016), suggesting that reaction time might be controlled separately of the movement preparation processes. To overcome this limitation, in this study we investigate the kinematic and geometrical effects that the shortening of the preparation time has on the performed trajectory.

The kinematic profiles of curved trajectories display multiple velocity peaks, possibly indicating that curved trajectories emerge from the composition of several kinematic trajectory units. Morasso and Mussa Ivaldi (1982) presented a model for trajectory formation, in which the more curved the movement is, the more it is likely to be composed of a larger number of time-overlapping kinematic motion primitives. Sosnik et al. (2015) demonstrated that in scribbling movements, subjects tend to continue movement production until a curved kinematic primitive is completed even if instructed to suddenly stop, indicating a feedforward planning mechanism which cannot be suddenly arrested during the generation of individual segments. In the context of curved movements, the underlying kinematic motion primitives might be straight, having a bell shape speed profile (Morasso and Mussa Ivaldi, 1982), as was suggested by the trajectory superposition scheme used to model trajectory modification (Flash and Henis, 1991; Henis and Flash, 1995), or curved (Sosnik et al., 2007; Polyakov et al., 2009b). Movement primitives may also have parabolic shapes as reported, for example, by Polyakov et al. (2009a) in a study of monkey scribbling movements. Notably, the underlying motion primitives may have more complex geometrical forms than those of straight or parabolic segments, as was indicated by a study of Hatsopoulos et al. (2007) showing that the patterns of neuronal firing of neurons in the primary motor cortex encode for motion fragments having rather complex geometrical shapes (Hatsopoulos et al., 2007). These studies have led us to consider the possibility that the shortening of preparation time may affect movement compositionality and the characteristics of kinematic motion primitives used to compose intentionally curved trajectories.

Movement planning may also involve an optimization process that determines the kinematic and geometric features of the composed motion primitives. Based on the concept of smoothness optimization as expressed by the minimum jerk model (Flash and Hogan, 1985), basic motion primitives may have either straight or parabolic shapes (although more complex shapes might also be employed). Specifically, point to point-minimum jerk trajectories having zero velocity and acceleration at the movement end-points follow straight hand paths. On the other hand, the paths of the minimum-jerk trajectories starting and ending at rest, but required to pass through a via point (Flash and Hogan, 1985), have geometrical shapes nearly indistinguishable from those of parabolic segments (Polyakov, 2006; Shpigelmacher, 2006). Hence, the jerk optimization model can successfully account for the geometrical shapes and temporal features of straight as well as curved obstacle avoidance movements.

Generating an optimal trajectory was recently shown to be a time-demanding process (Wong et al., 2016). This time demand could reflect a time-consuming process to construct an optimal control policy, or a time-consuming explicit planning of the entire trajectory. Regardless of the nature of the processes demanding an increase in reaction time under such scenarios, we predict that if movement preparation is hindered by the lack of time, subjects will initiate a sub-optimal movement plan. To study the optimization processes that occur during the planning phase, we chose an experimental paradigm called the “Timed Response paradigm” (Ghez et al., 1990), whereby subjects are forced to prematurely initiate an obstacle avoidance movement, immediately upon hearing the final beep in a series of four metronome-like beeps.

The goal of this study is to investigate the planning of intentionally curved trajectories by comparing their kinematic characteristics when generated under insufficient vs. sufficient preparation times. Using kinematic analysis, geometrical segmentation schemes and an optimization model, we find that when preparation time is not sufficiently long, subjects execute a sub-optimal initial trajectory and improve the performed motor plan “on the fly” by utilizing the segmented nature of the movement. Our observations show that the sub-optimality of the trajectories is reflected in a higher initial trial-by-trial variability and in larger deviations of the hand paths from the obstacle, but not in changes in the overall movement duration.

## Materials and Methods

### Participants

Fifteen right-handed subjects (7 males and 8 females, aged 22–31; mean age 26.1), naïve to the task and aims of the study, volunteered to participate in the experiment. The subjects reported being healthy without any known motoric limitations, an attention deficit disorder or dyslexia. All subjects signed a written informed consent form and received a small compensation for their participation in the study. The Sourasky center of medicine Review Board approved all procedures, which were in accordance with the Declaration of Helsinki.

### Experimental Setup

All recordings were conducted in a quiet room using a WACOM DTZ2100 tablet with a 142 Hz sampling rate and 500 × 1000 pixel per centimeter squared spatial sampling resolution. The tablet was synchronized with a PC using the Psychophysics Toolbox in MATLAB (Brainard, 1997; Pelli, 1997). Each subject was required to stand in front of the tablet, placed horizontally at a height of 85–94 cm, according to subject’s preference. Subjects were positioned such that the lateral end of the right clavicle was aligned with the tablet’s middle point. Additionally, the subjects’ feet positions were marked on the floor to allow them to keep a consistent posture throughout the experiment. Subjects held a touch screen stylus pen (WACOM) in their right hand. Their left hand remained free to the side of their body.

### Experimental Procedures

**Figure 1:**
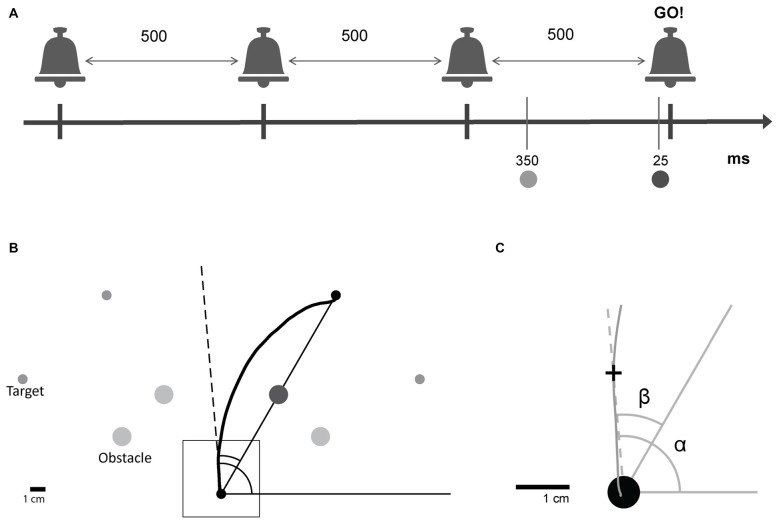
Experimental design. **(A)** Subjects were trained to leave the starting point on hearing a predictable auditory “go” signal, the last one in a sequence of four beeps. Beeps were separated by 500 ms. A target and an obstacle were presented in one of two preparation time conditions; Short: 25 ms or Long: 350 ms before the auditory “go” signal. **(B)** For each trial, one of four targets was presented with its corresponding obstacle. Subjects initiated obstacle-avoidance movements from the starting point towards one of the specified targets while avoiding the obstacle. **(C)** Magnification of the square shown in **(B)**. For each point along the trajectory, the angular position relative to the global reference frame (α, absolute angular position) and the angular position relative to the line connecting the starting point and the target (*β*, target-relative angular position) were calculated.

During the experiment, subjects were required to perform obstacle avoidance reaching movements on the tablet. The task was based on the Timed Response paradigm developed by Hening et al. (1988). Subjects were required to initiate an obstacle avoidance movement in synchrony with a predicted auditory “go” signal that was the last in a sequence of four beeps (see Figure 1A, experimental design). Movements started from a specified starting point. A trial was considered successful if the subject passed a 0.5 cm distance threshold from the center of the starting point, which was a circle with a radius of 0.3 cm, within a 300 ms time window, starting at the appearance of the “go” signal (fourth beep). An auditory feedback at the end of each trial informed the subjects whether their trial was successful.

Circular targets of radius 0.3 cm appeared at four possible locations (one target for each trial): 150°, 120°, 60° and 30° to the *x*-axis (referred to as target 1, 2, 3 and 4, respectively), distanced 15 cm from the starting point. A circular obstacle of radius 0.6 cm positioned exactly half way between the starting position and the target, at a distance of 7.5 cm from both (see Figure 1B), appeared at the same time with the target.

The target and obstacle were presented at one of two possible time points: either 25 ms or 350 ms before the “go” signal. The first condition forced subjects to initiate a movement using a shorter preparation time than the one naturally used, whereas the second condition allowed for enough preparation time for movement initiation. The exact preparation time values were chosen based on pilot studies that were conducted in the laboratory to detect the preparation times that elicit detectable behavioral effects. Preparation time is defined as the time between the presentation of the target and the initiation of the movement. Movement onset latency is defined as the time between the “go” signal and the initiation of the movement.

The experiment consisted of five sessions, each composed of 81 trials. The first session was dedicated to familiarize subjects with the requirements of the task (movement onset latency and obstacle avoidance), and to train the subjects to leave the starting point simultaneously with the “go” signal, with targets and obstacles presented precisely together with the “go” signal (0 ms time preparation time condition). The four following sessions included two sessions with short and two sessions with long preparation times. The order of the sessions was random.

The order and numbering of target appearance were counterbalanced, so that the probability for each target to appear after any previous displayed target was identical for all target pairs, including repetitions of the same target. Each session included five repetitions of each of the 4^2^ possible targets pairs.

### Data Analysis

Data were smoothed using a low pass filter with a cut off frequency of 10 Hz. For some analyses (parabolic fitting of data, and minimum-jerk prediction of trajectories; both requiring the calculation of high order derivatives of the data), data were smoothed by a low pass filter with a cut off frequency of 3.55 Hz. In order to ignore small oscillatory movements of the hand prior to movement initiation, movement onset and offset were defined using a velocity threshold (2% of maximal velocity). In the analysis of kinematic predictions based on the minimum jerk model (see “Materials and Methods”: “Prediction of Trajectory’s Suffix Based on Local Kinematics” Section) a more conservative threshold was applied (5% to 6% of maximal velocity). Accuracy was defined based on the probability of reaching a distance of less than 0.5 cm from the target. This distance was slightly larger than the size of the shown target (0.3 cm) to account for positional errors caused due to differences between the target presentation plane and the drawing plane.

### Angular Hand Positions throughout the Trajectory

Each trajectory was divided into 100 samples separated by intervals of equal arc lengths. We refer to these points as the arc length percentiles of the trajectory. At each percentile, we interpolated the Cartesian hand position. Subsequently, we extracted angular coordinates from each arc length percentile.

Two different angular coordinate frames were used for two types of analysis. First, for the analysis of reaching movements irrespective of their target, a common target-independent coordinate system was used; the absolute angular hand position, *α*, was defined for each hand position as the angle between the positive *x* axis and the line connecting the start point and hand position. Second, for target-specific analysis, a target-relative coordinate frame was defined. The target-relative angular hand position, *β*, was defined for each hand position as the angle between the line connecting the starting point and the target and the line connecting the starting point and the hand position (see Figure 1C).

### Bypassing Direction Analyses

In our design, a subject could bypass the obstacle from its right or left sides. Bypassing direction was determined for each trial according to the side of the maximal deviation from the line connecting the starting point and the target. Formally, we examined the target-relative angular position of maximal absolute size, *β*_max_ = *argmax_β_* |*β*|); indicating the maximum deviation from the line connecting the starting point and the target.

When *β*_max_ was positive it indicated a left bypassing, and when *β*_max_ was negative it indicated a right bypassing. Changing preparation time sometimes resulted in a change in the common bypassing direction. To overcome the variability induced by having two possible bypassing directions, in most of our analyses we used only movements that agreed with the subject’s dominant bypassing direction for the examined target, which was defined as the subject’s more frequently used direction for bypassing the examined target in the short preparation time condition.

### Statistical Analysis

A random effects analysis of variance (ANOVA) for repeated measures was used to detect significant differences in movement behavior among the different experimental conditions, using the MATLAB “anovan” command. Interaction effects were constrained to the a-priori contrasts of interest. A paired *t*-test was also used to compare behavioral patterns between conditions, using the MATLAB “ttest” command.

### Geometric Analysis

To examine how the geometric structure of the generated motions changes depending on the preparation time condition, we segmented the movement data based on the notion of geometric invariance. We used both straight lines and conic sections as geometric motion primitives (Guggenheimer, 1977) and identified segments whose geometric structure closely matched a straight line or a conic section, i.e., forming a parabola, a hyperbola, or an ellipse. We saw that most segments were either straight or parabolic. These well-studied primitives of motion are the geodesics of Euclidean and equi-affine geometries, respectively (Flash and Handzel, 2007). Parabolic segments account for the kinematics of scribbling motions in monkeys and were shown to correspond to transitions in cortical neural states recorded from monkeys (Polyakov et al., 2009a). Computationally, parabolas are also the solutions of jerk minimization with constant equi-affine speed (Polyakov et al., 2009b).

We defined straight segments as continuous portions of duration ≥0.07 s where the absolute Euclidean curvature did not exceed 0.05 cm^−1^. Consecutive segments with a gap of up to 0.025 s were united. We defined parabolic segments as continuous portions of duration ≥0.1 s, where the absolute value of equi-affine curvature did not exceed 1 cm^−4/3^ (calculated invariantly based on the work of Calabi et al., 1998). Consecutive parabolic segments separated by gaps of up to 0.025 s were united.

For each of these parabolic segments, we found a best fitting parabola and measured its parameters, determining the parabola’s orientation, its location and its geometric shape. The best fitting parabola was found in two steps. First, the best fitting parabola for each possible orientation was derived. Next, we chose the parabola that showed the best fit to the parabolic segment, across all possible orientations.

For each orientation angle, *γ*, we compared all parabolas whose symmetry axis made an angle *γ* with respect to the *y* axis. We first rotated the coordinate frame such that these parabolas had their symmetry axis parallel to the new *y* axis, by applying a rotation transformation (*x*_γ_, *y_γ_*) = *R_−γ_*(*x,y*). Here *x,y* are the original coordinates and *x*_γ_, *y*_γ_ are the new rotated coordinates aligned with the direction defined by the orientation angle, *γ*. Following rotation, all the oriented parabolas were described by 2nd order polynomials, $y_{\gamma }\left(x_{\gamma }\right)=ax_{\gamma }^{2}+\;bx_{\gamma }+c$. The best fitting parabola with the identified orientation was found by minimizing the least squared error $E=sum_{\text{i}}{\left(y_{\gamma_{\text{i}}}-{\hat{y}}_{\gamma }\left(x_{\gamma_{\text{i}}}\right)\right)}^{2}$, that is the sum of squared *y*_γ_ distances between the model parabola ${\hat{y}}_{\gamma }$ at each data point’s rotated coordinates *x*_*γ*i_ and measured values *y*_*γ*i_. We found the best fitting parabola for a given orientation by minimizing this error, using the MATLAB “polyfit” function, thus estimating for each *γ* the fitting error, *E*(*γ*), and its minimizing parameters *a, b, c*.

To find the optimal orientation, we minimized the error *E*(*γ*) as a function of the orientation angle, *γ*. To this end, we performed a nonlinear regression using the MATLAB “fminsearch” function. For each parabolic segment, the optimization process yielded the parameters *γ, a, b, c* defining the best fitting parabola. We report the results of the analysis of the rotation angle *γ* and the focal parameter: $P\;=\;\frac{1}{\left(2\sqrt{a}\right)}$, describing the orientation and curvature of the parabola, respectively. We also report analysis of the fitting error, *E*.

To compare the fitting errors between the two preparation time conditions, each segment was truncated to have a uniform length of 5 cm around its central point. There was no detectable presence of other types of conic segments such as elliptic (positive equi-affine curvature exceeding 1 cm^−4/3^) or hyperbolic (negative equi-affine curvature below −1 cm^−4/3^) in the data.

### Optimization Point of the Minimum Jerk Model

Based on the minimum jerk optimization model (Flash and Hogan, 1985), we developed and applied a method for predictive completion of a kinematic movement profile. For a specific trajectory drawn by the subject, $\vec{r}\left(t\right)$, we examined at each time point *t*_0_, what would be the predicted minimum jerk trajectory assumed to be initiated at a time *t*_0_ and having at this time position, velocity and acceleration vector values equal to those measured at that time, namely $\vec{r}=\vec{r}\left(t_{0}\right)$, $\vec{v}=\dot{\vec{r}}\left(t_{0}\right)$, $\vec{a}=\ddot{\vec{r}}\left(t_{0}\right)$ and $\;\vec{j}=\dddot{\vec{r}}\left(t_{0}\right)$. Using the local kinematics defined by these values and using the remaining movement duration from the time point *t*_0_ until movement end *T*, *T* – *t*_0_, as the movement duration for such a predicted trajectory, we predicted the suffix of the trajectory (i.e., the remaining part of the trajectory), following this time point, denoted by ${\vec{r}}_{t_{0}}\left(t\right)$, defined for *t*_0_ ≤ *t* ≤ *T*.

In a case that the original trajectory $\vec{r}\left(t\right)$ is a maximally smooth trajectory corresponding to a minimum jerk trajectory plan, then for any initial time point *t*_0_, the predicted suffix will overlap with the planned trajectory, ${\vec{r}}_{t_{0}}\left(t\right)=\vec{r}\left(t\right)$ for all *t* following *t*_0_. For an unplanned or a sub-optimal trajectory, the predicted continuation will most likely deviate from the planned trajectory, ${\vec{r}}_{t_{0}}\left(t\right)\;\neq \vec{r}\left(t\right)$ for all *t* following *t*_0_. Therefore, for any time point *t*_0_, the comparison of the predicted trajectory, ${\vec{r}}_{t_{0}}\left(t\right)$, to the actual trajectory, $\vec{r}\left(t\right)$, can be used to indicate whether the suffix starting at time *t*_0_ produced by the subject is well-planned; i.e., obeying the minimum jerk model while using the appropriate boundary conditions. Since this study focuses on planning a trajectory that ends at a pre-determined target, we compared the end-point of the predicted trajectory ${\vec{r}}_{t_{0}}\left(T\right)$ to the end-point of the actual recorded trajectory $\vec{r}\left(t\right)$. Each time point *t*_0_ was therefore assessed according to how well the predicted and measured end-points matched, by measuring the distance between the predicted and measured hand positions at time $T:d_{t_{0}}=d\left({\vec{r}}_{t_{0}}\left(T\right),\vec{r}\left(T\right)\right)$. The time point *t*_0_ for which these two end-points coincided (had a distance $d_{t_{0}}\leq 1\text{ cm}$) was called the optimal movement onset time *t_d_*. This time is the first one to correspond to the initiation of an optimal motor plan aimed at reaching the final movement target.

Technically, to discard possible biases due to differences in movement durations, we conducted analysis on the ratio between the optimal movement onset time *t_d_* and the trajectory’s total movement duration *T*. This ratio was named the normalized optimal movement onset time, defined as *t_nd_* = *t_d_*/*T*.

### Prediction of Trajectory’s Suffix Based on Local Kinematics

We used the minimum jerk model to predict a suffix ${\vec{r}}_{t_{0}}\left(t\right)$ of a movement trajectory.

In order to avoid relying on high order derivatives of noisy data, we preprocessed each trajectory by applying an optimization based filtering process, as suggested by Meirovitch et al. (2016) which overcomes the limitations of standard approaches such as Finite and Infinite Impulse Response filters.

To generate a filtered trajectory ${\vec{r}}_{f}\left(t\right)$ we minimized an optimization cost $C=\;\int {\left|\vec{j}\right|}^{2}\text{d}t+\lambda \;\int {\left|{\vec{r}}_{f}\left(t\right)-\vec{r}\left(t\right)\right|}^{2}\text{d}t$, where the filtered trajectory ${\vec{r}}_{f}\left(t\right)$ is having a geometric shape similar to that of the measured trajectory $\vec{r}\left(t\right)$ and is smoother than $\vec{r}\left(t\right)$ with respect to a minimum jerk criterion. Here λ is a constant representing a trade-off between smoothness and geometric resemblance to the measured trajectory.

Note that λ = 0 generates a minimum jerk trajectory (in the original sense of Flash and Hogan, 1985), and λ ≫ 0 generates a trajectory with a path practically indistinguishable from that of the measured trajectory *r*(*t*). After verifying that our results were insensitive to the value of λ, we set the value of λ to be λ = 30 s^−6^.

We predicted the trajectory’s suffix based on the movement’s measured duration and boundary conditions representing the local kinematics of the filtered trajectory ${\vec{r}}_{f}\left(t\right)$ at the time point *t*_0_, and at movement end time *T*. The two spatial coordinates, *x* and *y*, were treated separately, both complying with a 5th degree polynomials $x\left(t\right)=\sum_{i}c_{i}t^{i}$, $y\left(t\right)=\sum_{i}b_{i}t^{i}$ defined over the temporal region *t*_0_ ≤ *t* ≤ *T*. The coefficients *c*_*i*_, *i* = 0,…,5 defining *x*(*t*) were uniquely determined using six boundary conditions. Four equations defined the behavior at *t* = *t*_0_; *x*(*t*_0_) = *x*_0_, $\;\dot{x}\left(t_{0}\right)=v_{0}^{x}$, $\;\ddot{x}\left(t_{0}\right)=a_{0}^{x}$, $\;\dddot{x}\;\left(t_{0}\right)=j_{0}^{x}$ with values of *x*_0_, $v_{0}^{x}$, $a_{0}^{x}$, $j_{0}^{x}$ taken from the filtered trajectory ${\vec{r}}_{f}\left(t\right)$ at the time point *t*_0_. Two additional equations defined stopping at the end-point at *t* = *T*, setting speed and acceleration to zero, $\dot{x}\left(T\right)=0$, $\;\ddot{x}\left(T\right)=0$. We repeated the same process to find the coefficients *b_i_*, *i* = 0,…,5 defining *y*(*t*) using the same equations for the *y* coordinate. Notice that to predict a trajectory primarily based on the measured kinematics at time *t*_0_, we used non-symmetric boundary conditions with four equations describing the behavior at time point *t*_0_, compared to only two equations describing the behavior at end time *T*. This approach is different from the standard practice of using the minimum jerk model with symmetric boundary conditions of position, velocity and acceleration dictated at both the start and end-points (Flash and Hogan, 1985).

## Results

### Successful Task Performance under the Two Preparation Time Conditions

Before analyzing movement kinematics, we examined whether the differences between the conditions led to differences in task performance variables such as movement onset latency, accuracy, duration, peak velocity and path length. The average percentage of valid trials (i.e., trials in which the subjects managed to leave the starting point on time, passing a threshold distance of 0.5 cm from the center of the origin within 300 ms after the go cue) across all subjects was 89.4 (SE 1.8) (see “Materials and Methods” Section). The average percentage of valid trials was not affected by the preparation time condition (*F*_(1,29)_ = 1.48, *p* = 0.244). Trajectories that reached a distance of 0.5 cm from the center of the target were classified as accurate (see “Materials and Methods” Section). Trajectories showed high accuracy under the two preparation time conditions (93.5% success ±15.2 in the short and 95.85% success ±8.8 for long), with a relatively minor, but significantly larger, accuracy in the long preparation time condition (*F*_(1,119)_ = 6.32 *p* = 0.013).

### Movement Duration, Extent and Speed Were Not Affected by Preparation Time

The duration of movements (time between movement onset and offset, i.e., the first and last points whose speed was ≥2% of the peak velocity) was 0.68 s (SE 0.07), and did not change significantly between preparation time conditions and target conditions (*F*_(1,118)_ = 0.01, *p* = 0.92; *F*_(3,118)_ = 0.04, *p* = 0.99, respectively). The trials’ peak velocity was 46.69 (SE 4.86) cm/s, and this value also did not change significantly between the preparation time conditions and targets (*F*_(1,118)_ = 0.09, *p* = 0.76; *F*_(3,118)_ = 0.04, *p* = 0.99, respectively). The movement length, which was 17.8 cm (SE 0.4) did also not change significantly between the preparation time conditions and targets (*F*_(1,118)_ = 0.14, *p* = 0.71; *F*_(3,118)_ = 0.55, *p* = 0.64, respectively). Thus, subjects reached the target equally fast under the two preparation time conditions.

### Preparation Time Condition Did Not Affect Movement Onset Latency

**Figure 2:**
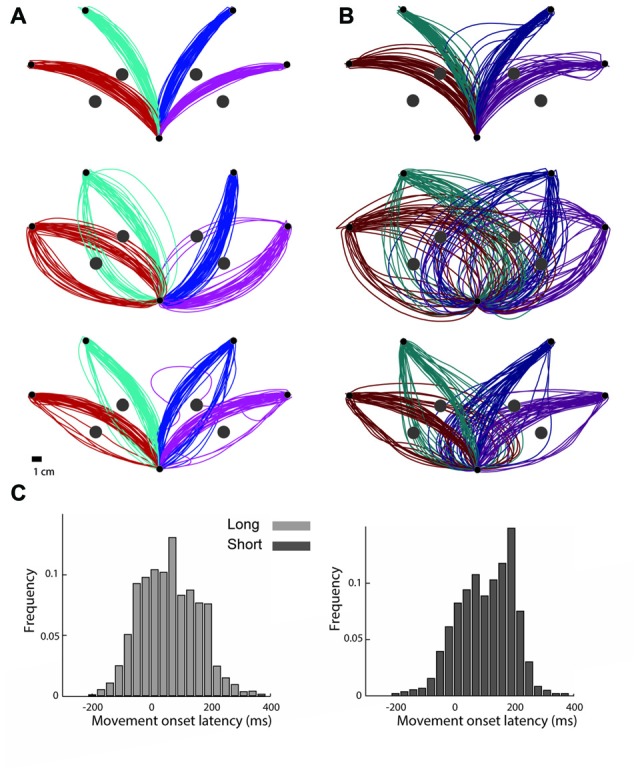
Trajectories from representative subjects. **(A)** Trajectories performed by three subjects under the long preparation time condition. Each inset shows trajectories taken from a single subject. The colors depict the targets for each trajectory. **(B)** Trajectories that the same subjects performed under the short preparation time condition. The colors depict the targets for each trajectory. **(C)** Onset latency times, measured from the “go” signal until the subject was reaching a speed of 5% of peak movement velocity, grouped across all subjects for movements performed under the long preparation time condition (left) and for movements performed under the short preparation time condition (right).

In order to further verify that the experimental manipulation indeed shortened preparation times and did not lead to a systematic increase in movement onset latency in the short preparation time condition, we compared movement onset latencies between the two preparation time conditions and found no effect (Figure 2C). We defined movement onset latency as the duration between the time of the “go” signal (the fourth beep) and a specified event signifying the beginning of the movement. Regardless of how this event was defined (2%, 5%, 7% and 10% of the total movement arc length, 2%, 5%, 7% and 10% of peak velocity, and crossing a velocity threshold of 5 or 10 cm/s), movement onset latencies were comparable across the two preparation time conditions (*p* > 0.38). Notably, the values of the movement onset latencies of the short preparation time condition (average of 144 ms according to a 5 cm/s velocity threshold) were comparable to movement onset latencies in other experiments using the timed response tasks where subjects were able to initiate accurate target selective point to point movements (Haith et al., 2016). This suggests that subjects in this study were able to perform target selection by the time they initiated the movement.

Interestingly, despite the lack of differences in movement durations and movement onset latencies and the small difference in accuracy, observing the trajectories that were performed under the two conditions, one can clearly appreciate the effect of preparation time on both the averaged trajectory and the trial-by-trial variability (Figure 2).

### Trajectories in Both Conditions Were Directed toward the Instructed Targets

One explanation for the observed results is that in the short preparation time condition subjects initially guessed the position of the target and only later made an online correction to eventually reach the indicated target, similarly to what happens in target jump experiments (Flash and Henis, 1991). To test this hypothesis, we examined the absolute angular positions at the beginning of the movement. We referred to the 10th percentile of the overall movement as an indicator for the feedforward part of the trajectory, and therefore as an estimator of the initial movement planning stage. We ran a two-way ANOVA for targets and preparation time conditions (four targets × two preparation time conditions) and found a significant target effect (*F*_(3,119)_ = 13.82, *p* < 0.0001), with no preparation time effect or interaction between target and preparation time condition factors (*F*_(1,119)_ = 0.2, *p* = 0.65 and *F*_(3,119)_ = 1.24, *p* = 0.30, respectively). Running ANOVA separately on each preparation time condition elicited a significant effect of target in both cases (*p* < 0.03). Lastly, to address the concern that in the short preparation time condition there was a considerable proportion of movements that did not show initial target-sensitivity (guessing), we split the short preparation time movements into two groups (according to movement onset latencies) and ran a two-way ANOVA with the effects of target and movement onset latency. We found a significant effect of target on the angular position (*F*_(3,119)_ = 3.59, *p* = 0.041) and no significant interaction between the movement onset latency group and target effects (*F*_(3,119)_ = 0.56, *p* = 0.65). These results demonstrate that despite the large variability in the trajectories that subjects made under the short preparation time condition, the trajectories were directed toward their indicated targets.

### Trial-by-Trial Variability of Relative Angular Hand Position Was Higher in the Short Preparation Time Condition

**Figure 3:**
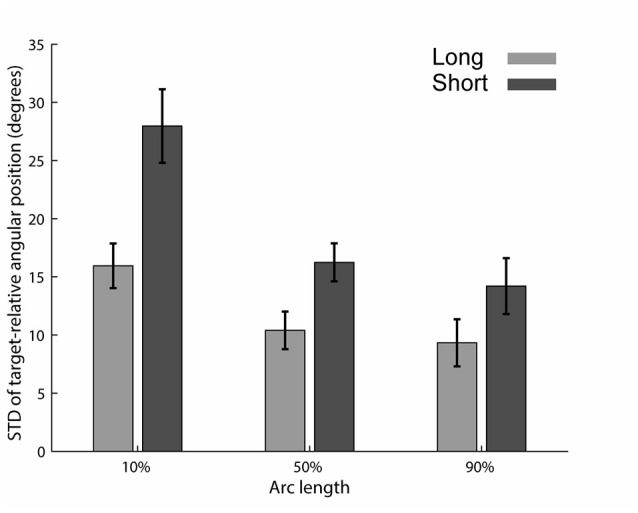
STD of target-relative angular positions. STD for the long (light gray) and short (dark gray) preparation time conditions. Relative angular hand positions were calculated in relation to each target’s axis (see Figure 1). Mean and standard error across subjects are shown.

We were first interested in examining if reducing the preparation time reduces the consistency of the trajectories across trials. To test this hypothesis, we compared the trial-by-trial variability along the trajectory in both preparation time conditions. We calculated the standard deviation of the target-relative angular positions (Figure 1B) at three points along the trajectory (10th, 50th and 90th percentiles of the arc length, Figure 3), separately for each subject. Only movements having the same obstacle bypassing direction were selected for this analysis (see “Materials and Methods”: “Bypass Directions Analyses” Section). Paired *t*-test on the three points of interest revealed a significant difference in STD levels between the short and long preparation time conditions (*p* < 0.001 for the 10th and 50th percentiles and *p* = 0.026 for the 90th percentile). In order to compare the distributions of angular positions directly, we ran Levene’s test on the data of each subject separately at movement onset (10th percentile of movement length). We found that most subjects (12/15) had a significantly larger variability of the relative angular position for the short preparation time compared with the long preparation time conditions (*p* < 0.05).

### Shortening Preparation Time Affects Feedforward Planning

**Figure 4:**
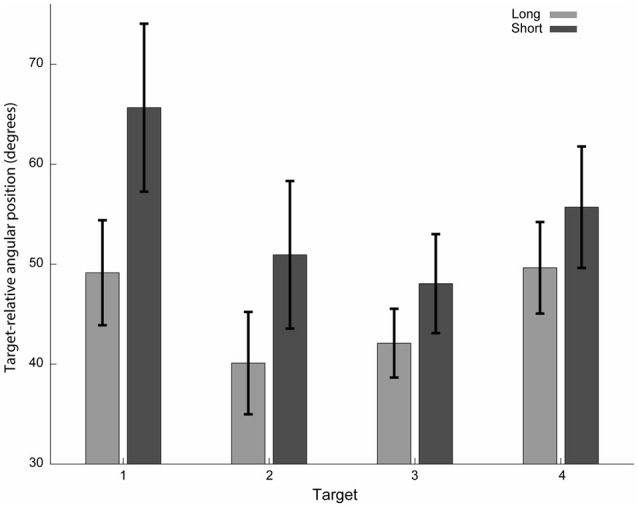
Target-relative angular hand positions at movement onset. Initial direction of the short preparation time condition’s trajectories (dark gray) deviated more from the line connecting the starting point and the target than for the long preparation time condition (light gray). The absolute values of angular hand positions with respect to a line connecting the starting point and the target are presented. Only trajectories with the dominant bypass direction for each target are included. Mean and standard errors across subjects are shown.

In addition to the increased variability, there were also marked differences in the averaged trajectories that subjects performed (Figures 2, 4), suggesting that the plan itself was also sensitive to the preparation time. We focused our analysis on the initial part of the movement to emphasize the contribution of preparation time to the feedforward plan of the movement. We first hypothesized that the initial direction of the movement will be sensitive to preparation time. We calculated the mean target-relative angular position at the onset of the movement (10th percentile of the movement length). As predicted, the deviation from the line connecting the starting point and the target during the short time condition was larger than that for the long time condition. A two-way ANOVA test, modeling target and preparation time conditions (four targets × two preparation time conditions), revealed a preparation time condition effect (*F*_(1,119)_ = 6.53, *p* = 0.012).

Note that this difference in initial conditions was not associated with differences in total duration, peak speed, and length of the trajectories. We suggest that this apparent discrepancy reflects the local nature of this effect. When the entire trajectories were analyzed, the differences in relative angular positions at movement onset was not apparent.

### Geometric Decomposition of Movements

**Figure 5:**
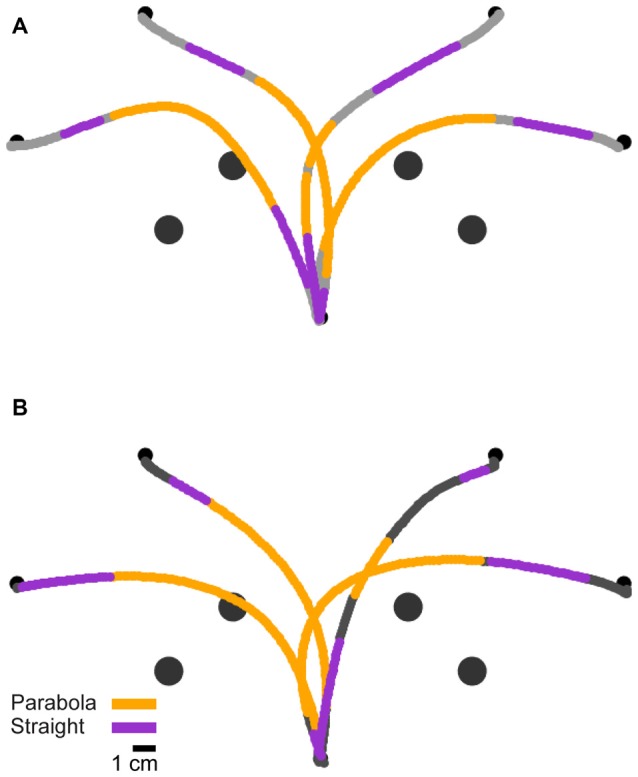
Geometric segmentation. **(A)** Trajectories of a typical subject from the long preparation time condition, segmented into parabolic (orange) and straight (magenta) segments. **(B)** Trajectories of the same subject from the short preparation time condition.

The previous sections showed support for the existence of different movement plans in the two preparation time conditions. We next asked if the observed effect of preparation time on the execution of the movement would also be evident from the geometrical properties of the trajectory. Accumulating results show that complex trajectories are composed of sub-units, or primitives, among them geometric primitives (Flash and Hochner, 2005; Polyakov et al., 2009a; Giszter, 2015). The hypothesis of this section is that preparation time will affect the composition of the trajectory from motion primitives, as can be inferred based on the geometric properties of the trajectories and their segmentation. Based on our inspection of the data, we chose to focus our trajectory segmentation analysis on straight and parabolic segments, defined based on the Euclidean and equi-affine curvature profiles of the trajectories (see Figure 5 and “Materials and Methods”: “Geometric Analysis” Section). The motivation behind using equi-affine geometrical analysis stems from previous reports showing that various curved human trajectories have constant equi-affine velocities and that parabolic segments (which have 0 equi-affine curvature) serve as basic kinematic primitives in the construction of human and monkey movements (Polyakov et al., 2009b).

### Parabolic Segments Appear Later When Preparation Time Is Short

**Figure 6:**
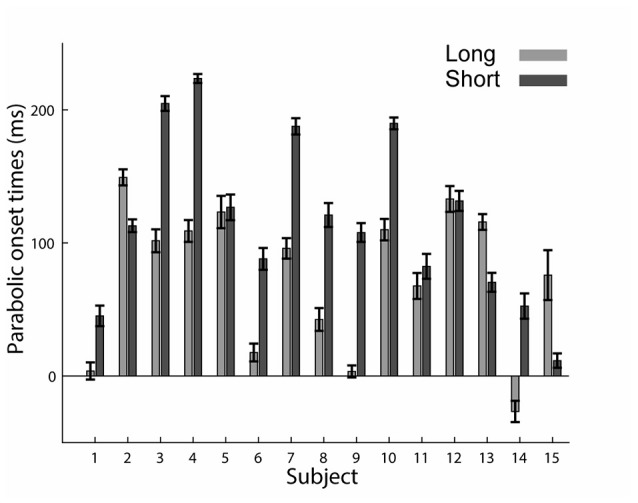
Onset of the parabolic segments across subjects. Mean and SE of durations from the “go” signal to the onset of parabolic segment. For most subjects, the parabolic segments started later in the short preparation time condition.

Based on the decomposition of the trajectories into an initial parabolic segment which is followed by a straight segment, we hypothesized that if the parabolic elements represent a component that is planned as a unit stroke, it may appear later in the trajectory in trials where subjects had a shorter preparation time. In other words, when preparation time is shorter, the parabolic segment will appear later. Indeed, we found that in the short preparation time condition, the onset of the parabolas (computed with respect to the “go” signal) came on average 42 ms later than in the long preparation time condition (75 ms ± 4 in the long and 117 ms ± 4 in the short preparation time conditions (*p* = 0.016)). Figure 6 presents the onset of the parabolic segments for each subject. Note that in subject 14, the onset of the parabolic segment in the long preparation time condition is negative due to a consistent initiation of the movement before the “go” signal. Furthermore, when examining the time of initiation of the parabolic segments with respect to the movement onset (and not to the “go” signal), we found that it is negatively correlated with movement onset latency. Thus, whenever the movement onset latency was shorter, subjects initiated their parabolic segment later during their movement. A linear fit of movement onset latency and the duration between movement onset and parabolic onset, performed on a subject-by-subject basis, resulted in linear coefficients that were always negative (*p* = 0.001 for the short preparation time condition and *p* = 0.002 for the long preparation time condition). These results should be taken cautiously given the poor fits of the model (*R*^2^ of −0.20 (SE 0.046) for the short condition, and −0.15 (SE 0.039) for the long preparation time condition). This regression analysis was feasible due to the natural variability in onset latencies (see Figure 2C).

### Modeling of the Parabolic Segments Revealed Preparation Time Effects

Despite the differences in the onset of the parabolic segments, arc-lengths and durations of the parabolic segments were not affected by the preparation time condition (*F*_(1,29)_ = 0.17, *p* = 0.679 for segments’ durations, *F*_(1,29)_ = 0.0023, *p* = 0.962 for segments’ arc lengths). To furtherly examine the differences between the parabolic segments performed under the two preparation time conditions; we fitted parabolas to the parabolic segment. The parabolas matched each parabolic segment closely, with a root mean square error of 0.006 (SE 0.001) cm and 0.012 (SE 0.003) cm for the parabolic segments of long and short preparation times, respectively. Interestingly, the quality of those fits was poorer for the short preparation time condition (*F*_(1,29)_ = 8.47, *p* = 0.011). This difference cannot be attributed to differences in the size of the segments since the segments were truncated to a uniform length of 5 cm around their center point, to obviate any modeling bias.

We next investigated the parameters used to describe the parabolas in the two preparation time conditions. The rotation angle *γ* of the parabolic segment represents the angle of the symmetry axis of the best fitting parabola with respect to the *y* axis. This *γ* angle showed a significant target effect (*F*_(3,119)_ = 160.79, *p* < 0.001) but not a preparation time condition effect (*F*_(1,119)_ = 0.02, *p* = 0.902) nor target and preparation time interaction (*F*_(3,119)_ = 2.03, *p* = 0.115). We examined the focal parameter P of the parabola that expresses the width of the parabola. We found a significant preparation time (*F*_(1,119)_ = 12.99 *p* < 0.0001), and target (*F*_(3,119)_ = 7.73, *p* < 0.001) effects, but no interaction between these effects (*F*_(3,119)_ = 0.93, *p* = 0.431) on the focal parameter.

To summarize, compared to the long preparation time condition, the parabolas in the short time condition had a significantly smaller focal parameter, indicating that their underlying parabolas were more curved.

### Later Onset of Target-Specific Optimal Trajectories in the Short Preparation Time Condition

**Figure 7:**
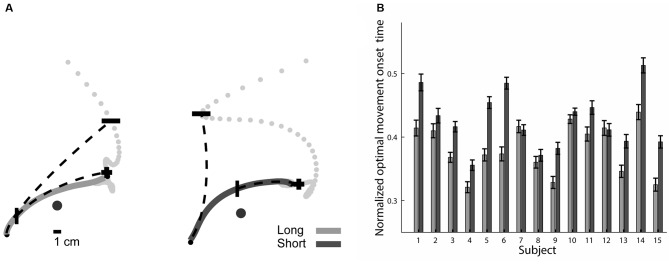
Optimal movement onset times. **(A)** Optimal movement onset points marked on top of exemplary trajectories for long (left) and short (right) preparation time conditions. Trajectories are depicted by solid lines. For each trajectory, we show two examples of predicted trajectories (dashed line), one is based on the movement onset (small black circle) and the other is based on the optimal movement onset point (“|”). The end-points of all possible predicted trajectories are also shown (light gray dots). The predicted trajectory starting at movement onset ends at a predicted end-point (“−”) that is far from the actual movement end, while the predicted trajectory starting at the optimal movement onset point (“|”) ends at a predicted end-point (“+”) near the actual movement’s end. **(B)** Mean and SE of normalized optimal movement onset times for all subjects. These times occurred at an earlier stage in the long compared to the short preparation time conditions.

The last section pointed toward a possible mechanism for refining the trajectories that subjects made under the short preparation time condition during their execution. In this section we follow this direction and examine at what point the trajectories are becoming optimized given their total duration and their end-point target, under the assumption that the outcome of a complete trajectory planning process is a maximally smooth movement plan. We applied the minimum jerk model (Flash and Hogan, 1985) at each point along the trajectory and calculated the point where the predicted trajectory will terminate (see “Materials and Methods”: “Prediction of Trajectory’s Suffix Based on Local Kinematics” Section). As the subject’s pen moved along his trajectory, the predicted movement end-point approached the target of the movement, until these points coincided. The position of the pen that predicted movement end-point was labeled as the optimal movement onset point (Figure 7A). The normalized optimal movement onset time, the time from movement onset to the point where the trajectory was a target-specific optimized trajectory, normalized by the total movement duration, indicates the completion of the optimization of the subject’s motion plan (Figure 7B). In the short preparation time condition, the normalized optimal movement onset time occurred later than in the long preparation time condition (*F*_(3,119)_ = 3.42, *p* = 0.020, Figure 7B), providing further support for an online optimization mechanism. The optimal plan onset times were not significantly correlated with the movement onset latencies; the linear coefficient was −0.093 (SE 0.048) for the short preparation time condition (not significantly different than 0, *p* = 0.075) and 0.001 (SE 0.026) for the long preparation time condition (not different from 0, *p* = 0.983).

### Correspondence between Target Prediction and Segmentation Analyses

Finally, we asked if the results of the decomposition analysis, pointing to a later initiation of a parabolic segment in the short condition, and the results of the kinematic prediction analyses, pointing to a later optimization of the trajectory in the short condition, are related. We therefore examined if the onset times of the parabolic segment and the optimal movement onset times are similar. We found that the optimal movement onset times came significantly later in the trajectory (*p* < 0.001 in both preparation time conditions) and that the parabolic onset and optimal movement onset times did not show a consistent relationship within subjects (*R*^2^ < 0.05 in each of the subjects, in both conditions). These results suggest that the two reported descriptions do not reflect the same planning aspect, and demonstrate that the planning continues even after the onset of the parabolic segment.

## Discussion

We investigated the planning processes of curved trajectories by measuring the effect of preparation time shortening on the executed trajectory. By manipulating subject’s preparation time, we were able to find qualitative and quantitative differences between the performed trajectories. We argue that these differences reflect time-consuming performance optimization processes that take place both during the preparation time and during movement execution. Our results show that the trajectories formed under the short preparation time condition were more variable and showed a larger initial angular deviation from the obstacle. When examining the geometrical characteristics of the trajectory, we found that the curved segments from which it is composed show worse fit to a parabola for the short preparation time condition and started later. The onset of the parabolic segment was correlated with movement onset latency in both preparation time conditions. Furthermore, a minimum jerk optimization model showed that the point in time where the short condition trajectories became optimal appeared later.

We suggest that these differences indicate that in the short preparation time condition, while subjects could possibly select their endpoint target, they did not have sufficient time to plan their trajectory, and therefore took advantage of the ability to continue planning their trajectories after movement initiation, refining their trajectories on the fly. This conjecture is consistent with a recent report demonstrating that in a reaction time penalty task subjects reduce their reaction time and refine their trajectories on the fly (Orban De Xivry et al., 2017).

### Increased Initial Trajectory Variability in the Short Preparation Time Condition

Variability in performance may stem from different stages in the planning and execution processes of a motor behavior: visual detection of target location, action selection and trajectory planning and execution. We found that the initial trial-by-trial variability was higher in the short preparation time condition, indicating that preparation time affects the consistency of the executed trajectories. The increased variability could be a result of differences in the action selection process. In fact, since in our design the same target always appeared with the same obstacle, subjects could link the selection and planning processes, and not go through a *de novo* motor planning of each trajectory. Thus, in the short preparation time condition, when subjects did not have enough time to complete the process of target detection and selection, they could have guessed their target end-point and its linked trajectory. Nevertheless, the fact that the angular position of the trajectories at movement onset showed sensitivity to the end-point target at the short preparation time condition supports the argument that at least part of the variability is not an outcome of guessing but rather of incomplete path and trajectory specification. This claim is also supported by the fact that the movement onset latencies in the short condition are compatible with previous studies that showed intact target selection processes under comparable latencies (Haith et al., 2016).

We therefore argue that the increased variability in the short condition is an outcome of a poorer trajectory planning process. In other words, we argue that the source of the increased variability in the short preparation condition is downstream from the target selection stage and is likely to be the result of an insufficient time for planning the required curved trajectory. This result suggests that performance variability does not depend only on movement speed (Schmidt and Lee, 1999), target difficulty (Fitts, 1954) or skill (Müller and Sternad, 2004; Shmuelof et al., 2012) but also on preparation time and complexity of the trajectory. Thus, we suggest that when the performed trajectories are more complex, for instance, when maneuvering around obstacles, planning involves a time-consuming trajectory specification stage as well. The effect of increased complexity on computation time is consistent with a recent report about an increase in reaction time for obstacle avoidance movements when compared to point to point movements (Wong et al., 2016). Our results are complementary to these results, as they address the characteristics of the trajectory planning process, using the timed response paradigm. To summarize, our findings suggest that when subjects are required to execute composite trajectories, the planning that takes place before movement onset contributes to the quality of trajectory’s execution and motion performance.

### On the Fly Compensation for Target-Insensitive Initial Plan

Our results suggest that the accumulation of information for motor planning continues after movement initiation. The delayed onset of the parabolic segment in the short preparation time condition provides a possible mechanism for integrating the new information into the trajectory. Interestingly, the fact that the optimization time came after the onset of the parabolic segment, suggests that the process of compensating for the initial sub-optimal plan, due to insufficient preparation time, is likely to be a continuous process.

Ongoing trajectory specification is also shown in paradigms involving decision making tasks, whereby motor commands are updated during movement execution based on the accumulation of evidence (Resulaj et al., 2009; Friedman et al., 2013). We speculate that the same mechanism may be utilized for the refinement of the motor plan.

Our results, supporting the existence of continuous motor planning, are also consistent with results of motor imagery studies which demonstrated that motor imagery is a time-consuming process whose durations (Jeannerod and Decéty, 1995; Roth et al., 1996; Rodriguez et al., 2008, 2009; Guillot et al., 2012) and kinematic profiles (Papaxanthis et al., 2012; Karklinsky and Flash, 2015) are closely correlated with those of executed movements. Under the hypothesis that motor imagery is equivalent to motor planning (Jeannerod and Decéty, 1995; Jeannerod, 1995), the fact that motor imagery is not instantaneous and evolves through time provides another argument in favor of our conjecture that movement planning is a continuous process.

### Isochrony through Control of the Time Dimension

Despite the robust effects of preparation time on initial variability and on the motor plan, the ability of subjects to reach the target at the end of the movement was hardly impaired. This suggests that subjects utilized feedback correction mechanisms to refine their movements on the fly. Interestingly, even though the task did not enforce a specific movement duration, subjects did not modulate the duration or the maximum speed of their movements to compensate for initial errors. This result, which is consistent with the isochrony principle (Viviani and Flash, 1995), suggests that the selection of movement duration might be explicitly controlled, or could possibly be dictated by different considerations. Some finite horizon optimization models (e.g., the optimal feedback control model (Todorov and Jordan, 2002) or the minimum jerk (Flash and Hogan, 1985) or the minimum variance models (Harris and Wolpert, 1998) assume that the total movement duration is a-priori prescribed. In alternative models which include the infinite horizon modeling approach or the “cost of time” model (Berret and Jean, 2016), movement time or a function of it is either explicitly included in the objective function (e.g., Tanaka et al., 2006) or is selected to give an integrated minimum cost for the chosen objective function (Huh et al., 2010). Movement durations predicted by optimization models in which they are not explicitly prescribed (i.e., “the cost of time” and the infinite horizon models), are not expected to obey the isochrony principle. On the other hand, our observations are consistent with affine invariance predicting full isochrony of the trajectory plans (Bennequin et al., 2009). Regardless of the mechanistic explanation of our findings, they support the importance of the isochrony principle, by showing that the motor system, despite the enforced shortening in reaction time, does not exploit modulations of the overall movement duration. This is surprising, because the movement is composed of several segments.

### Individual Strategies for Complex Tasks

Of a particular interest is the fact that subjects presented a large variety of individual preferences with respect to their initial directions (for some examples, see Figure 2). These preferences of initial direction cannot be explained by a recency effect since we controlled for the targets history (Jax and Rosenbaum, 2007). Previous studies have found that preferences in the initial direction of the movement depend on the posture of the arm (Ghilardi et al., 1995). Hence, a possible explanation for this phenomenon is mobility considerations, i.e., that the dynamics of the arm make some movement directions easier than others. Nevertheless, in a recent pilot experiment that we conducted, we tested the effect of subject’s position with respect to the tablet, and found that subjects were consistent in their preferences even when standing to the right or to the left of the tablet, suggesting that the idiosyncratic preferences cannot be fully explained by the dynamics of the arm. These preferences are therefore likely to point to the fact that the optimization processes of complex behaviors allow for more pronounced inter-subject differences. This conjecture is supported by recent results showing individual characteristics of motion under loose task constraints during a ball catching task (Cesqui et al., 2012) and a bimanual polyrhythmic task (Park et al., 2013).

### On the Theoretic Relationship between the Geometric Segmentation and the Minimum Jerk Model

Our modeling approaches pointed toward two behavioral effects that were driven by preparation time shortening. The geometrical analysis showed that the parabolic segment started later in the short preparation time condition, and the kinematic modeling showed a later optimization of the trajectory towards the target. Nevertheless, the onset times of the parabolic path and the optimal movement onset times did not correlate and did not coincide in time; the optimal movement onset times occurred after the onset of the parabolic segments. Both results show that when subjects have sufficient preparation time they execute a smooth trajectory earlier than when enforced to initiate a trajectory before they are fully ready. This could reflect the existence of a motor plan before movement initiation. The fact that the optimization time occurred later suggests that the planning continues following the onset of the parabola. The adjustment of the plan after the onset of the parabola could be done either by an abort-replan mechanism, but more likely, by a superposition of another primitive on top of the ongoing motion (Flash and Henis, 1991; Henis and Flash, 1995). The optimization time may therefore reflect the onset of the last superimposed motion primitive. The corrective primitive towards the end of the movement could be either a straight path having a minimum jerk temporal profile or a more complex kinematic primitive.

## Conclusion

We report that reducing preparation time of obstacle avoidance trajectories to four possible targets from ~490 ms to ~170 ms did not affect target selection process but led to an increase in trial-by-trial variability and to a modification of the mean trajectory. Our results therefore demonstrate the effect of preparation time on trajectory optimization. When optimization is not completed before movement onset, subjects delay the implementation of the central parabolic segment, and initiate a smooth trajectory towards the target later in the trajectory. We therefore suggest that subjects utilize the segmented nature of curved movements to refine their motor plan on the fly. These observations suggest that movement preparation is only part of the movement planning process that continues throughout complex movements, allowing on the fly optimization of the motor plan. Our results point toward a balance between the preference of the motor system to specify the trajectory before its initiation and the ability of the motor system to initiate sub-optimized trajectory rapidly and refine it on the fly.

## Author Contributions

DK, MK, TF and LS designed the experiment. DK and MK collected the data. DK, MK, YM and LS analyzed the data. MK, YM, TF and LS wrote the manuscript.

## Conflict of Interest Statement

The authors declare that the research was conducted in the absence of any commercial or financial relationships that could be construed as a potential conflict of interest.
